# Disseminated intravascular coagulation

**DOI:** 10.1186/s40560-025-00794-y

**Published:** 2025-06-06

**Authors:** Satoshi Gando, Marcel Levi, Cheng-Hock Toh

**Affiliations:** 1 Department of Acute and Critical Care Medicine, Sapporo Higashi Tokushukai Hospital, N33, E14, Higashi-Ku, Sapporo, Japan; 2 Division of Acute and Critical Care Medicine, Department of Anesthesiology and Critical Care Medicine, Hokkaido University Faculty of Medicine, Sapporo, Japan; 3 Department of Vascular Medicine, Amsterdam University Medical Center, Amsterdam, The Netherlands; 4 Department of Medicine, University College London Hospitals NHS Foundation Trust, Cardiometabolic Program-NIHR UCLH/UCL BRC, London, UK; 5 Department of Clinical Infection, Microbiology and Immunology, University of Liverpool, Liverpool, UK; 6 Department of Haematology, Liverpool University Hospitals NHS Foundation Trust, Liverpool, UK

**Keywords:** Cell death, Complement, Disseminated intravascular coagulation (DIC), Histone, Hypoxia, Hemorrhage, Innate immunity, Inflammation, Management, Organ dysfunction

## Abstract

**Background:**

Disseminated intravascular coagulation (DIC) is characterized by systemic coagulation activation, anticoagulation pathway impairment, and persistent fibrinolysis suppression, resulting in widespread microvascular thrombosis, followed by hemorrhagic consumption coagulopathy and multiple organ dysfunction syndrome. This article aimed to provide a comprehensive and updated DIC overview.

**Main body:**

The International Society on Thrombosis and Hemostasis provides definitions, underlying disorders, diagnostic algorithms, and management guidelines for DIC. Two clinical features of DIC are hemorrhagic consumption coagulopathy, characterized by oozing and difficult-to-control bleeding, and microvascular thrombosis, leading to dysfunctions in multiple vital organs. Histones derived from cellular damage play central roles in the innate-immune-based coagulation model, comprising the initiation, amplification, propagation, and reinforcement phases, which, if dysregulated, develop into DIC. Thus, the innate immune-mediated pathogenic pathways in DIC have become clear. Cell death, damage-associated molecular patterns (including histones), crosstalk between hypoxic inflammation and coagulation, and the serine protease network (comprising coagulation and fibrinolysis, the Kallikrein–Kinin system, and complement pathways) play major roles in DIC pathogenesis. Conversely, these pathogenic pathways and DIC synergistically contribute to organ dysfunction, leading to poor prognoses. Effective DIC management requires treating the underlying condition, along with substitution therapies and, in some cases, antifibrinolytics. Anticoagulant use has been extensively debated; however, the selection of optimal target patients could optimize their application and improve patient outcomes in the near future.

**Conclusions:**

This review provides an updated overview of DIC, aiming to help readers understand various aspects of DIC today.

## Introduction

Disseminated intravascular coagulation (DIC) is characterized by systemic coagulation activation, anticoagulation pathway impairment, and persistent fibrinolysis suppression, resulting in widespread microvascular thrombosis followed by hemorrhagic consumption coagulopathy and multiple organ dysfunction syndrome (MODS) [1]. Therefore, DIC, a thrombohemorrhagic disorder, is recognized as a life-threatening condition that affects critical illness prognoses.

A review by MacKay in 1969 summarizing the past two decades of progress in DIC research described the definition, underlying disorders, clinical features, pathogenesis, and treatments of DIC, building the foundation for the development of DIC studies [2, 3]. In 1972, Colman proposed three conditions that cause DIC: platelet activation with coagulant phospholipid release, tissue injury with coagulation activation, and endothelial injury [4]. In addition, the laboratory value thresholds for DIC diagnosis were established for the first time. Developments in cytokine research during the 1990s brought a new paradigm of mutual interplay between coagulation and inflammation, followed by the establishment of new cytokine-based pathogenetic pathways in DIC [5, 6]. The establishment of DIC diagnostic criteria by the International Society on Thrombosis and Hemostasis (ISTH) in 2001 enabled the selection and validation of a homogenous patient population for therapeutic interventions, further advancing DIC research [7]. In the 2000s, neutrophil extracellular traps (NETs) and histones (derived from nuclear contents of damaged cells) were shown to affect DIC pathogenesis through immunothrombosis [8–10], with dysregulated immunothrombosis promoting pathological thrombosis in DIC [10]. Histones and cell-free DNA comprising NET components are further involved in MODS pathogenesis in DIC [11, 12]. In addition to NETosis, necrosis and regulated cell death, such as pyroptosis, have been identified as major sources of tissue factors that are important DIC initiators [13]. The coagulation, kallikrein–kinin system (KKS), and complement pathways forming the serine protease network have mutual interactions among them, and the network also plays pivotal roles in DIC pathogenesis [14, 15].

Inflammation and coagulation in innate immunity are tightly coupled to maintain homeostasis against the insults by delimiting and fixing the insult-mediated tissue injury [5, 16, 17]. Dysregulation of these systems leads to DIC associated with bleeding and MODS, which may permit DIC to be referred to as Dysregulated Immune Coagulation. This review aimed to provide a comprehensive and updated overview of DIC that aims to help readers understand various aspects of DIC today.

### Epidemiology

Differences in the study period, country, diagnostic criteria, diagnosis setting (emergency department, intensive care unit [ICU], or ward), and various underlying disorders have made it challenging to accurately evaluate DIC epidemiology. In addition, some studies applied the International Classification of Disease code to identify DIC, which may weaken the accuracy of DIC diagnosis. However, several large cohort studies published between the early 1990s and the 2020s provide valuable insights [18–25] (Table 1). DIC incidence is estimated at 20–30 cases per 100,000 people across various settings, showing a mild decline [20, 21, 23]. Although DIC remains a devastating condition with high mortality rates, the mortality rate has declined by > 50% during the 1990s to < 50% in the 2010s, with two large cohort studies further reporting a 41.8% (2010) to 34.7% (2021) decline over 10 years [24, 25]. The most common underlying DIC disorders are infection/sepsis, followed by cardiac arrest, obstetric complications, and trauma in Denmark, and solid cancers, hematologic malignancies, trauma, and obstetric complications in Japan [23–25]. In the Denmark cohort, hematological malignancies were excluded; however, the reason for the differences in the incidence of underlying disorders between the two countries remains unclear.

**Table 1:** DIC epidemiology

Years	Diagnosis	Design	Setting/center	Number	Incidence (%)	Mortality (%)	Outcome	References
1992	JMHW	JMHW^a^	Ward/multicenter	123,231	1.04	65.2	Hospital	[18]
1998	JMHW	JMHW^a^	Ward/multicenter	108,792	1.87	56.0	Hospital	[19]
2004–2010	ISTH	Retrospective	ICU/single center	8089	26.2–18.6^b^	58.0–45.0^c^	Hospital	[20]
2006	ISTH	Retrospective	ICU/single center	1461	18.0^d^	76.0	Hospital	[21]
2010–2012	ICD-10	DPC database	Various/multicenter	34,711	NA	48.4–43.9^e^	Hospital	[22]
2013–2020	ISTH/JAAM	CDR database	Various/multicenter	2565	33.1–24.0^f^	35.0–41.3^f^	30 days	[23]
2010–2017	ICD-10	DPC database	Various/multicenter	325,327	NA	41.8–36.1^g^	28 days	[24]
2010–2021	ICD-10	DPC database	Various/multicenter	443,098	NA	41.8–34.7^h^	28 days	[25]

*CDR* Central Denmark Region Hospital Laboratory Database, *DPC* Japanese Diagnosis Procedure Combination Inpatient Database, *ICD-10* International Classification of Disease Tenth Revision, *ICU* intensive care unit, *ISTH* International Society on Thrombosis and Hemostasis, *JAAM* Japanese Association for Acute Medicine, *JMHW* Japanese Ministry of Health and Welfare, *NA* not applicable

^a^Nationwide epidemiological survey by the JMHW

^b^Incidence values for 2004 and 2010 are per 100,000 population

^c^Change in incidence between 2004 and 2010; *p* = 0.38

^d^Incidence adjusted per 100,000 population (age-adjusted)

^e^Change in mortality between 2010 and 2012; *p* < 0.001

^f^Incidence values for 2013 and 2020 are age- and sex-adjusted per 100,000 population; the study reported a significant average annual percent change in incidence without corresponding improvement in mortality

^g^Change in mortality between 2010 and 2017; *p* < 0.001

^h^Change in mortality between 2010 and 2021; *p* < 0.001

### Definition, diagnosis, and underlying conditions

#### Definition

The ISTH defined DIC as “an acquired syndrome characterized by the intravascular activation of coagulation with loss of localization arising from different causes. It can originate from and cause damage to the microvasculature, which, if sufficiently severe, can produce organ dysfunction.” [7]. A key aspect of this definition is that tissue factor-induced thrombin generation extends beyond the initial site of insult or damage to microvascular endothelial cells, resulting in microvascular fibrin thrombosis associated with organ dysfunction. Systemic coagulation activation also consumes coagulation factors and platelets, contributing to hemorrhagic consumption coagulopathy. Furthermore, the ISTH emphasized that generalized inflammatory responses in DIC lead to vasodilation and tight junction loss in endothelial cells, resulting in capillary leakage associated with shock [7].

#### Diagnosis

The ISTH and the Japanese Association for Acute Medicine (JAAM) have proposed two prospectively validated DIC diagnostic algorithms [7, 26, 27] (Table 2). The gold standard for evaluating the diagnostic characteristics of the ISTH and JAAM scoring systems was the agreement of DIC diagnosis by two experts of DIC and the established Japanese Ministry of Health and Welfare (JMHW) DIC diagnosis, respectively, ensuring the robustness of both systems. The relationships between both scoring systems have been validated and characterized that (1) the JAAM scoring system has good sensitivity, while the ISTH system has good specificity for diagnosing DIC, (2) JAAM DIC often progresses to ISTH DIC as severity increases, and (3) both systems effectively diagnose DIC and predict poor prognoses [28, 29]. To maintain diagnostic specificity, the ISTH scoring system recommends not calculating scores without clinical conditions listed in the scoring system, while the JAAM scoring system includes a list of conditions requiring careful exclusion [7, 27]. Both algorithms recommend repeated score measurements to improve diagnostic accuracy.

**Table 2:** DIC diagnostic algorithm

	ISTH DIC	JAAM DIC	Points
Underlying disease	Table attached^a^ Yes, proceed No, do not use this algorithm	Table attached^a^	–
Clinical conditions that should be carefully ruled out	–	Table attached	–
Platelet counts (10^9^/L)	–	< 80 , or > 50% decrease/24 h	3
	< 50	–	2
	≥ 50, < 100	≥ 80< 120, or >30% decrease/24 h	1
FDP (µg/mL) or fibrin-related markers (such as soluble fibrin/fibrin degradation products)	Strong increase	≥ 25	3
	Moderate increase	–	2
	–	≥ 10, < 25	1
Prothrombin time (sec or ratio)	≥ 6 s	–	2
	≥ 3, < 6 s	≥ 1.2	1
Fibrinogen (g/mL)	< 100	–	1
SIRS criteria	–	≥ 3	1
Criteria-positive points	5 points^b^	4 points	–

*DIC* disseminated intravascular coagulation, *FDP* fibrin/fibrinogen degradation products, *ISTH* International Society on Thrombosis and Hemostasis, *JAAM* Japanese Association for Acute Medicine, *SIRS* systemic inflammatory response syndrome

^a^Both scoring systems provide table showing the lists of underlying disease of DIC

^b^If ≥ 5, repeat scoring daily; if < 5, suggestive (not affirmative) for non-overt DIC, repeat the next 1–2 days

#### Underlying conditions

The ISTH summarized research from the past several decades, identifying eight conditions underlying DIC development [7]. In 2020, the list was revised based on evidence linking specific conditions to DIC [30]. The strength of the evidence was classified as “high” or “low.” High-level evidence includes “certain” when studies show pathologic microthrombi in small vessels and “probable” when DIC is confirmed by established diagnostic criteria. Low evidence applies to conditions with < 10 reported cases and no clear association with DIC. Table 3 summarizes underlying DIC conditions with high-level evidence, categorized as certain or probable.

**Table 3:** Clinical conditions associated with DIC with a high level of evidence (certain/probable)

Main subgroup	Specific causes
Severe infection	
	*Bacterial sepsis* Viral infection Protozoal infection Systemic mycosis
Tissue damage	
Due to external insult	*Major trauma* (blunt trauma, penetrating trauma or tool/weapon-mediated trauma) Extensive burn Hyperthermia (such as heat stroke) Hypothermia Hypoxia/ischemia due to asphyxia or drowning
Due to internal insult	Acute pancreatitis Hypoxia/ischemia due to cardiac impairment (such as cardiac arrest, cardiogenic shock)
Solid tumor	
Gastrointestinal cancer	*Esophageal, gastric, and pancreatic cancers* Hepatic cell carcinoma
Genitourinary cancer	*Prostate cancer*
	Lung cancer Breast cancer
Hematologic neoplasia	
Acute leukemia	*Acute myeloid leukemia* (such as acute promyelocytic, acute monocytic leukemia) Acute lymphoblastic leukemia
Myeloproliferative disorder	Chronic myelogenous leukemia (blast crisis) Multiple myeloma
Biological and chemical agent	
	*Snake bite* Transfusion of incompatible blood Transplant rejection
Vascular disease	
	*Aneurysm* Giant hemangioma (such as Kasabach–Merritt syndrome)
Pregnancy complication	
	*Placental abruption* Post-partum hemorrhage (associated with uterine atony, placenta previa and/or adhesion, uterine rupture or hematoma of the birth canal, uterine inversion) Amniotic fluid embolism HELLP syndrome Preeclampsia and eclampsia Retained dead fetus Acute fatty liver
New-born complication	
	*Perinatal asphyxia* Respiratory distress syndrome Congenital anatomical abnormalities (such as cardiovascular and gastrointestinal system) Necrotizing enterocolitis

Italics indicate the most common conditions in each group

*DIC* disseminated intravascular coagulation, *HELLP* hemolysis, elevated liver enzyme, and low platelet count

### Phenotypes

#### Overt and non-overt

Overt DIC is defined as a stressed and decompensated hemostatic system, often at an irreversible stage, while non-overt DIC involves a stressed but compensated hemostatic system [7]. To identify non-overt DIC as hemostatic dysfunction starting to decompensate, the ISTH proposed a diagnostic framework distinct from the overt DIC scoring system, in which dynamic changes in platelets and coagulation parameters and sensitive molecular markers were used [7]. In non-overt DIC, systemic thrombin generation may be neutralized by the anticoagulant systems on the regulated endothelium or remain insufficient to meet overt DIC criteria. Daily repeated DIC score measurements are mandatory for monitoring progression from non-overt to overt DIC.

#### Controlled and uncontrolled

Endothelial regulatory anticoagulation networks comprising protein C/thrombomodulin, antithrombin, and tissue factor pathway inhibitor (TFPI) are transiently overridden in controlled DIC but swiftly restored when the causal factors are resolved [7]. In contrast, these factors and their regulatory networks are overwhelmed in uncontrolled DIC, resulting in disruption in the microvasculature [7]. In controlled DIC, the endothelium remains activated, such as in abruptio placentae and early trauma phase, where rapid hemostasis through surgical procedures and hemorrhagic shock prevention facilitates re-equilibration. In uncontrolled DIC, the endothelium is injured, switching from an anti- to a procoagulant state and from pro- to antifibrinolytic, such as in sepsis and late trauma phase, where DIC therapeutic interventions are required.

#### Thrombotic and fibrinolytic

DIC is typically a prothrombotic phenotype characterized by coagulation activation, insufficient anticoagulation, and persistent fibrinolysis suppression by plasminogen activator inhibitor 1 (PAI-1) [1]. However, DIC with a fibrinolytic phenotype is characterized by the coexistence of thrombotic DIC with fibrin-independent systemic pathological hyperfibrinogenolysis under a single clinical condition [31, 32]. Pathological insults always lead to thrombin-induced fibrin formation; therefore, DIC with a fibrinolytic phenotype can be rephrased as the coexistence of DIC and systemic pathological hyperfibrin(ogen)olysis. The mechanisms underlying this condition include (1) acceleration of tissue-type plasminogen activator (t-PA) release from endothelial Weibel–Palade bodies due to hypoxia [33–35] or from organ storage pools after destruction [35–38]; (2) plasminogen-to-plasmin conversion acceleration via the expression of urokinase-type plasminogen activator (u-PA) receptor [38–41]; and (3) plasminogen-to-plasmin conversion acceleration via annexin A2 and S100A10 heterotetramer expression on tumor cells [42–44] or endothelial cells due to thrombin, hypoxia, and hyperthermia [45–47]. Table 4 presents the mechanism-related underlying conditions of DIC with a fibrinolytic phenotype. Further details are reviewed elsewhere [31].

**Table 4:** Underlying conditions of DIC with a fibrinolytic phenotype

Mechanism	Underlying condition
t-PA release from endothelial cells	
Ischemia/hypoxia, shock, hypoperfusion	Trauma and traumatic shock Cardiac arrest and resuscitation Postpartum critical bleeding Asphyxia and drawing
Heat stress	Heat stroke
t-PA release from storage pool	
Brain	Isolated traumatic brain injury
Malignant solid tumor	Prostate cancer Breast cancer Lung cancer
Conversion of plasminogen to plasminrevise headings.	
Cell surface expression of u-PA receptor	Prostate cancer
Cell surface expression of Annexin A2/S100A	Acute promyelocytic leukemia
Endothelial expression of Annexin A2/S100A	
Thrombin and hypoxia	Trauma and traumatic shock Cardiac arrest and resuscitation Postpartum critical bleeding Asphyxia and drawing
Heat stress	Heat stroke

*DIC* disseminated intravascular coagulation, *t-PA* tissue-type plasminogen activator, *u-PA* urokinase-type plasminogen activator

### Clinical features and pathology

#### Hemorrhagic consumption coagulopathy

Systematically generated thrombin and plasmin account for consumptive decreases in platelets, fibrinogen, and factors II, V, and XIII, followed by decreases in factor X (FX) due to rapid clearance [32]. Thrombin potentiates its production by activating FXI, and FXIa subsequently activates FIX, enhancing consumption. Thrombin also provides positive feedback on FVIII; however, FVIII is usually increased in DIC as an acute phase reactant released from Weibel–Palade bodies along with von Willebrand Factor [48, 49]. FVII is also consumed, as observed in an in vivo model of non-overt DIC and in patients with DIC [48, 50]. Proteolytic cleavage of a disintegrin-like metalloproteinase with thrombospondin type 1 motif 13 (ADAMTS13) by thrombin, plasmin, and neutrophil elastase is increased in DIC, resulting in high levels of ultra-large von Willebrand factor, accelerating platelet count reduction [51]. In addition, platelets in DIC are hyperactivated and exhausted with reduced aggregation ability [52]. Consequently, platelet dysfunction and the consumptive decrease in platelets and coagulation factors contribute to bleeding in patients with DIC. Clinical manifestations of consumptive hemorrhage in DIC often present as oozing and difficult-to-stop bleeding at the mucosa (nasal, gastrointestinal, and genitourinary), skin (purpura [petechiae, ecchymosis, and suggillation] and hemorrhagic bullae), injury site, vessel puncture site, and surgical field. Cerebral bleeding remains a serious complication.

#### Microvascular thrombosis

Microvascular platelet and fibrin thrombosis have been confirmed by clinical, autopsy, and experimental findings in the brain, lung, liver, kidney, heart, pancreas, adrenal gland, spleen, pituitary gland, and gastrointestinal tract [2, 3, 53, 54]. Hemorrhagic necrosis and thrombus formation in medium-to-large-sized vessels are common autopsy findings. Isolated traumatic brain injury, trauma, and traumatic shock typically present as DIC with a fibrinolytic phenotype [31]. In the hyperacute phase in these conditions, characterized by exacerbated fibrinolysis, microvascular fibrin thrombosis has been observed in vital organs, highlighting the coexistence of massive thrombin formation and excessive fibrinolysis [55–57]. Considerable evidence supports the association between thrombosis and organ dysfunction [54]: (1) ischemic tissue necrosis correlated with thrombosis extent, quantity, and duration [58, 59]; (2) intravascular thrombosis was associated with secondary neuronal cell death [60, 61]; and (3) site-inactivated FVIIa prevented fibrin thrombus formation in the lungs and kidney, improving organ injury [62]. However, inhibition of FXa downstream of tissue factor/FVIIa, blocked DIC without preventing organ damage, suggesting that other factors participate in DIC-induced organ dysfunction [54, 63]. This point is discussed in a later section.

Visible signs of microvascular thrombosis include bilateral peripheral acral cyanosis and extremity necroses [64]. In addition, organ dysfunction signs in the brain (consciousness disturbance, convulsion, and bleeding), cardiovascular (myocardial infarction and thromboembolism), liver (hepatic failure and jaundice), lung (hypoxemia and respiratory distress syndrome), gastrointestinal (mucosal necrosis, ulceration, and intestinal ischemia), kidney (acute tubular necrosis, cortical necrosis, oliguria, and hematuria), and adrenal insufficiency (hemorrhagic necrosis) are observed in DIC [53]. Adrenal insufficiency in DIC, caused by bilateral hemorrhagic necrosis of the adrenal glands due to *Neisseria meningitidis* infection, is known as Waterhouse–Friderichsen syndrome or fulminant purpura and usually presents with MODS [65]. Some patients with DIC exhibit fibrin thrombus-induced hemolytic anemia with red cell fragmentations, which should be differentially diagnosed from thrombotic microangiopathy [53, 66].

### Coagulation pathways

#### Protease cascade and cell-based coagulation

Tissue factor initiates coagulation by forming a complex with FVII, known as the tissue factor/activated FVII (FVIIa) complex [1]. This complex activates factors IX and X, forming complexes with FVIIIa and FVa to create the tenase (FIXa/FVIIIa/Ca^2+)^ and prothrombinase (FXa/FVa/Ca^2+^) complexes, respectively. The coagulation cascade comprises a series of proteolytic reactions involving serine proteases that form a central protease, thrombin (FIIa), from prothrombin (FII) activation by the prothrombinase complex. Thrombin converts fibrinogen into fibrin, activating FXIII to form cross-linked fibrin. Thrombin also provides positive feedback on factors V, VIII, and XI, potentiating thrombin generation. Although FXIIa activates FXI, both are involved in the KKS, contributing to inflammation, fibrinolysis, and complement pathway activation [14, 31, 67, 68]. Coagulation cascade activation is controlled by three major anticoagulant pathways in the endothelium: protein C and thrombomodulin regulate FVa and FVIIIa, while TFPI and antithrombin (both connected to glycocalyx heparan sulfates) regulate the tissue factor/FVIIa complex and FXa, and thrombin and factors VIIa, IXa, Xa, XIa, and XIIa, respectively.

The traditional cascade model of coagulation, comprising the extrinsic and intrinsic pathways, has been replaced by a cell-based hemostasis model that incorporates mutual cellular interactions with clot formation [69]. Activated platelets expressing phosphatidylserine provide a platform for coagulation initiation, amplification, and propagation. Exposure of tissue factor-bearing cells, such as endothelial cells, to tissue factors, initiates coagulation, generating a priming amount of thrombin that activates platelets via protease-activated receptors that amplify coagulation. The coagulation propagates, forming a tenase complex on activated platelets and a final prothrombinase complex to produce large amounts of thrombin (thrombin burst). Thrombin production is localized to the site of injury; however, uncontrolled coagulation due to disturbed anticoagulant systems gives rise to DIC [69].

#### Innate immune-based coagulation

NETs and their components (histones and cell-free DNA) play crucial roles in linking inflammation and coagulation as part of the host response to maintain homeostasis [10]. The link between these factors in the cell-based homeostasis model has emerged and developed into a convergent coagulation model involving inflammation and innate immune activation [70, 71]. Histones are central to this process.

##### Initiation

Histones expose tissue factors in the circulation by disrupting endothelial integrity through cytotoxicity, disturbance of junctional protein expression, and cell death [9, 72–75]. They also induce tissue factors in monocytes and endothelial cells [73, 76]. Tissue factor decryption is followed by phosphatidylserine exposure and protein disulfide isomerase expression in activated platelets and endothelial cells [77, 78]. In addition to the tissue factor/FVIIa complex activation of FX into FXa, histones directly bind to FXa as a substitute for FVa, forming an alternative prothrombinase complex that generates thrombin independently of phospholipids, further accelerating prothrombinase activity away from cell surface [79].

##### Amplification

Histones amplify coagulation through platelet and endothelial cell activation and degranulation. They activate platelets via toll-like receptor 2 (TLR2) and TLR4 [80–82] or ionophore induction and calcium influx [82], leading to phosphatidylserine exposure, P-selectin expression, and polyphosphate release [81, 83]. Furthermore, histones induce endothelial phosphatidylserine exposure and von Willebrand factor release [78, 84, 85].

##### Propagation

Phosphatidylserine exposure on platelets and endothelial cells promotes coagulation by coupling the tenase and prothrombinase complexes. In addition, histones contribute to clot firmness by covalently crosslinking to fibrin via FXIIIa and by non-covalently thickening fibrin fibers, increasing resistance to t-PA [86–88].

##### Reinforcement

Histones reinforce fibrin clot formation through procoagulant NET induction, inflammatory cytokine expression, and upregulating of selectins and adhesion molecules [10, 70, 89, 90]. They also regulate anticoagulant pathways. Histones regulate thrombomodulin-mediated protein C activation by directly binding to and reducing thrombomodulin antigen and mRNA levels to enhance thrombin generation [84, 91]. NET-bound neutrophil elastase degrades and inactivates TFPI, thrombomodulin, and antithrombin [10, 13]. Histone competitively inhibits antithrombin activity by binding to glycosaminoglycans on proteoglycans [13, 92].

**Fig. 1:**
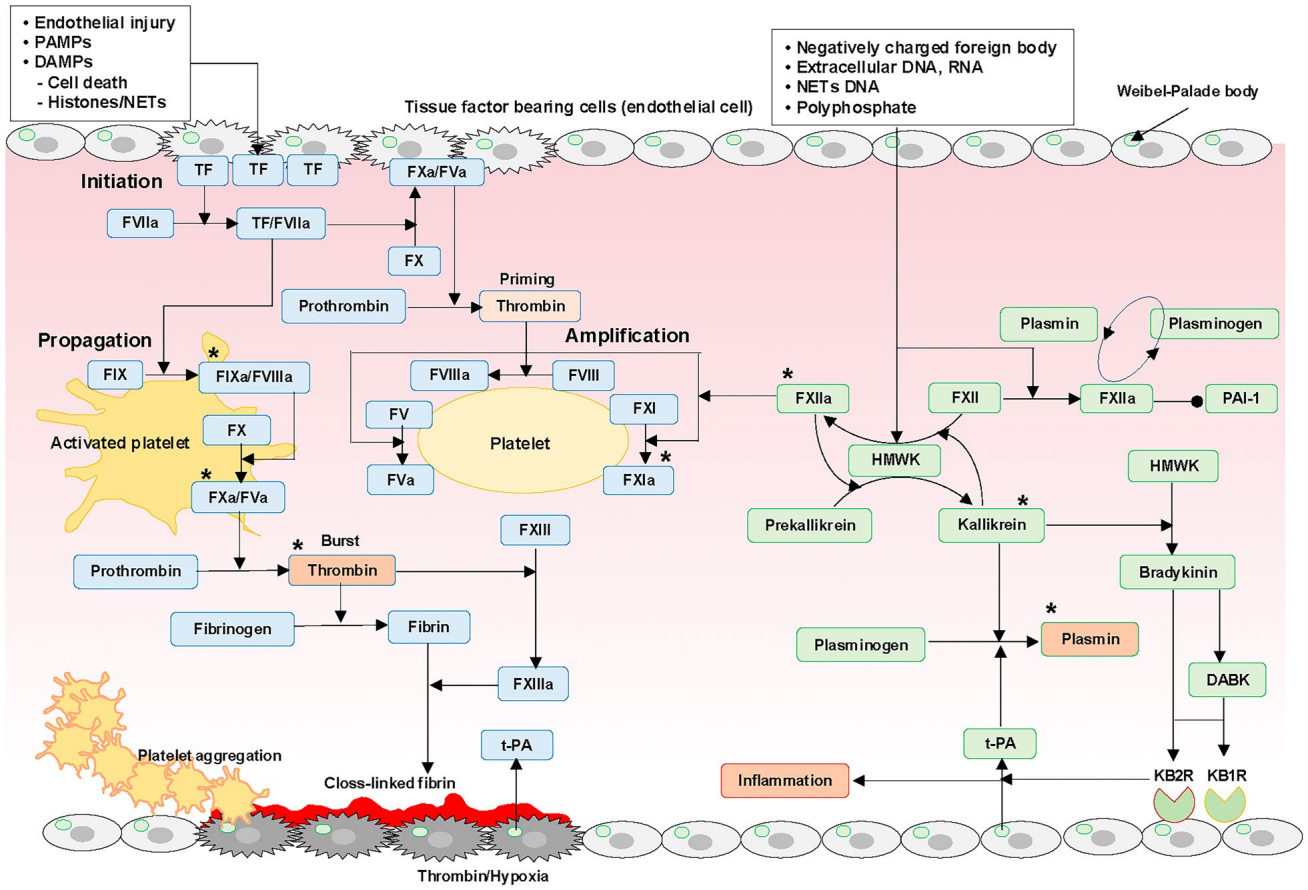
Coagulation pathways and the Kallikrein–Kinin system. Injury-, PAMP-, or DAMP-mediated tissue factor exposure or expression in the circulation from tissue factor-bearing cells (figure assumes endothelial cells) triggers coagulation initiation, amplification, and propagation phases. In the innate immune-based coagulation model, histones play central roles in all three phases and reinforce coagulation by physiologically suppressing anticoagulation systems. Thrombin and tissue hypoxia under fibrin clots stimulate t-PA release from Weibel–Palade bodies in the endothelium, inducing fibrinolysis. Various DAMPs activate FXII, initiating KKS, where FXIIa, kallikrein, and t-PA-released Weibel–Palade bodies via bradykinin binding to KB2R convert plasminogen into plasmin. Bradykinin and its active metabolite DABAK induce inflammatory cytokine expression via KB2R and KB1R, respectively. Thus, coagulation, fibrinolysis, and inflammation are tightly coupled through the FXIIa and KKS axis, and serine proteases (*) have been well known to activate complement pathways, as depicted in Fig. 5. *DABAK* des-Arg^9^-bradykinin, *DAMPs* damage-associated molecular patterns, *HMWK* high-molecular weight kininogen, *KB1R, KB2R* bradykinin B1 and B2 receptors, *KKS* Kallikrein–Kinin system, *NETs* neutrophil extracellular traps, *PAI-1* plasminogen activator inhibitor-1, *PAMPs* pathogen-associated molecular patterns, *TF* tissue factor, *t-PA* tissue-type plasminogen activator

**Fig. 2:**
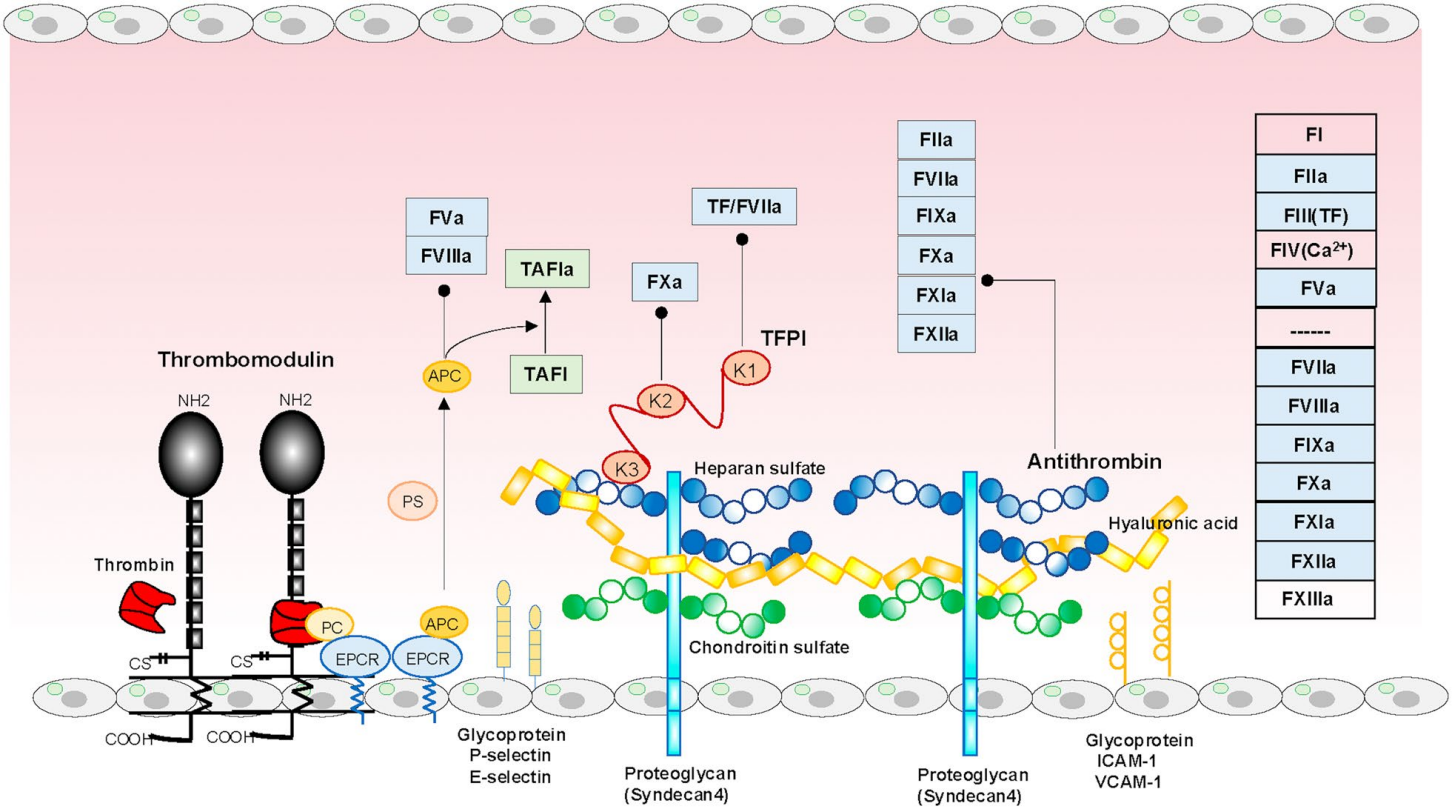
Anticoagulation pathways and glycocalyx. Glycocalyx comprises glycoproteins, proteoglycans, and glycosaminoglycans. Thrombin binds to endothelial surface glycoprotein, thrombomodulin, via its epidermal growth factor-like domains 5 and 6. The formed complex activates protein C binding to EPCR to generate APC. The APC complexes with PS, degrading FVa and FVIIIa and accelerating thrombin-mediated TAFI activation to TAFIa, resulting in coagulation and fibrinolysis inhibition. TFPI comprises three Kunitz-type serine protease inhibitor domains (K1, K2, and K3), binding to glycosaminoglycan heparan sulfate through the K3 domain. K1 and K2 inhibit tissue factor/FVIIa complex and FXa, respectively. Proteoglycan syndecan has four subtypes. Syndecan 4 complexes with heparan sulfate and chondroitin sulfate, facilitating antithrombin binding to syndecan 4 heparan sulfate, thereby inhibiting factors IIa, VIIa, IXa, Xa, XIa, and XIIa. In summary, all coagulation factors except FI (fibrinogen), FIV (Ca^2+^), and FXIII (transglutaminase) can be controlled by the three anticoagulant systems. In addition, the anti-inflammatory properties of APC, TFPI, and antithrombin are well known. Glycoproteins such as selectins, ICAM-1, and VCAM-1 are upregulated or induced upon insults, mediating leucocyte rolling, tethering, adhesion, and transmigration across the endothelium. *APC* activated protein C, *EPCR* endothelial protein C receptor, *ICAM-1* intercellular adhesion molecule-1, *PC* protein C, *PS* protein S, *TAFI* thrombin-activatable fibrinolysis inhibitor, *TAFIa* activated TAFI, *TF* tissue factor, *TFPI* tissue factor pathway inhibitor, *VCAM-1* vascular cell adhesion molecule-1

The tight interplay between innate immune inflammation and coagulation is essential for the balance between physiological immunohemostasis and pathological immunothrombosis in the response to injury. However, dysregulation of these systems results in excessive inflammation and thrombin generation associated with overly reinforced (or insufficient) anticoagulant pathways, easily giving rise to DIC [1, 10, 13, 70]. This connection is supported by clinical and in vivo evidence [93–95]. Figures 1 and 2 summarize coagulation and anticoagulation processes, respectively.

## Pathogenic pathways

### Cell death

Various insults release pathogen-associated molecular patterns (PAMPs) and damage-associated molecular patterns (DAMPs), leading to innate immune inflammatory reactions. Both PAMP and DAMP signals are efficiently converted into new DAMPs through cell death mechanisms, suggesting that DAMPs may be major contributors to innate immune inflammation [96]. Cell death comprises accidental cell death (necrosis), regulated cell death (necroptosis, pyroptosis, and apoptosis [programmed cell death]), and active NETosis [97]. All cell death, including apoptosis, lead to the release of DAMPs, such as histones, nuclear and mitochondrial DNA (nDNA and mitDNA), and high-mobility group box 1 (HMGB1), which are involved in DIC pathogenic pathways [11–13, 96, 98].

#### Necrosis and NETosis

The bidirectional involvement of necrosis and NETosis in DIC pathogenesis has been extensively reviewed [12, 70]. Necrosis-released histones trigger neutrophils to release NETs (webs of nuclear DAMPs [histones and nDNA)), depositing proteases, such as elastase and myeloperoxidase. Histones and NETs synergistically enhance inflammation and coagulation via platelet activation and tissue factor expression in monocytes and endothelial cells. Anticoagulant pathways are impaired by the downregulation, functional inhibition, and degradation of thrombomodulin, protein C, TFPI, and antithrombin. The cytotoxic effects of histones on endothelial cells result in endothelial injury, further impairing anticoagulation systems. Fibrinolysis inhibition also occurs through the action of histones and NETs. Moreover, in vivo*,* histone infusion models reproduce the processes and DIC development associated with organ dysfunction [79, 95].

#### Regulated cell death

Pyroptosis contributes to DIC pathogenesis via inflammasome-dependent pathways, particularly the nucleotide-binding oligomerization domain (NOD)-like receptor (NLR) family pyrin domain-containing protein3 (NLRP3) inflammasome [99, 100]. Canonical and noncanonical inflammasome activation-induced macrophage pyroptosis triggers tissue factor-positive microvesicles release through gasdermin D (GSDMD)-dependent pore formation with subsequent calcium influx associated with phosphatidylserine exposure and plasma membrane rupture by nerve injury-induced protein 1 (NINJ1) [96, 101, 102]. Tissue factor release is associated with systemic thrombosis and lethality; moreover, GSDMD depletion prevents tissue factor-induced DIC during endotoxemia [101, 102]. Another study supported these results, showing that the GSDMD inhibition-mediated reduction in tissue factor activity reduced levels of thrombin and antithrombin complex, D-dimer, PAI-1, fibrinogen, organ dysfunction, and survival rate [103].

**Fig. 3:**
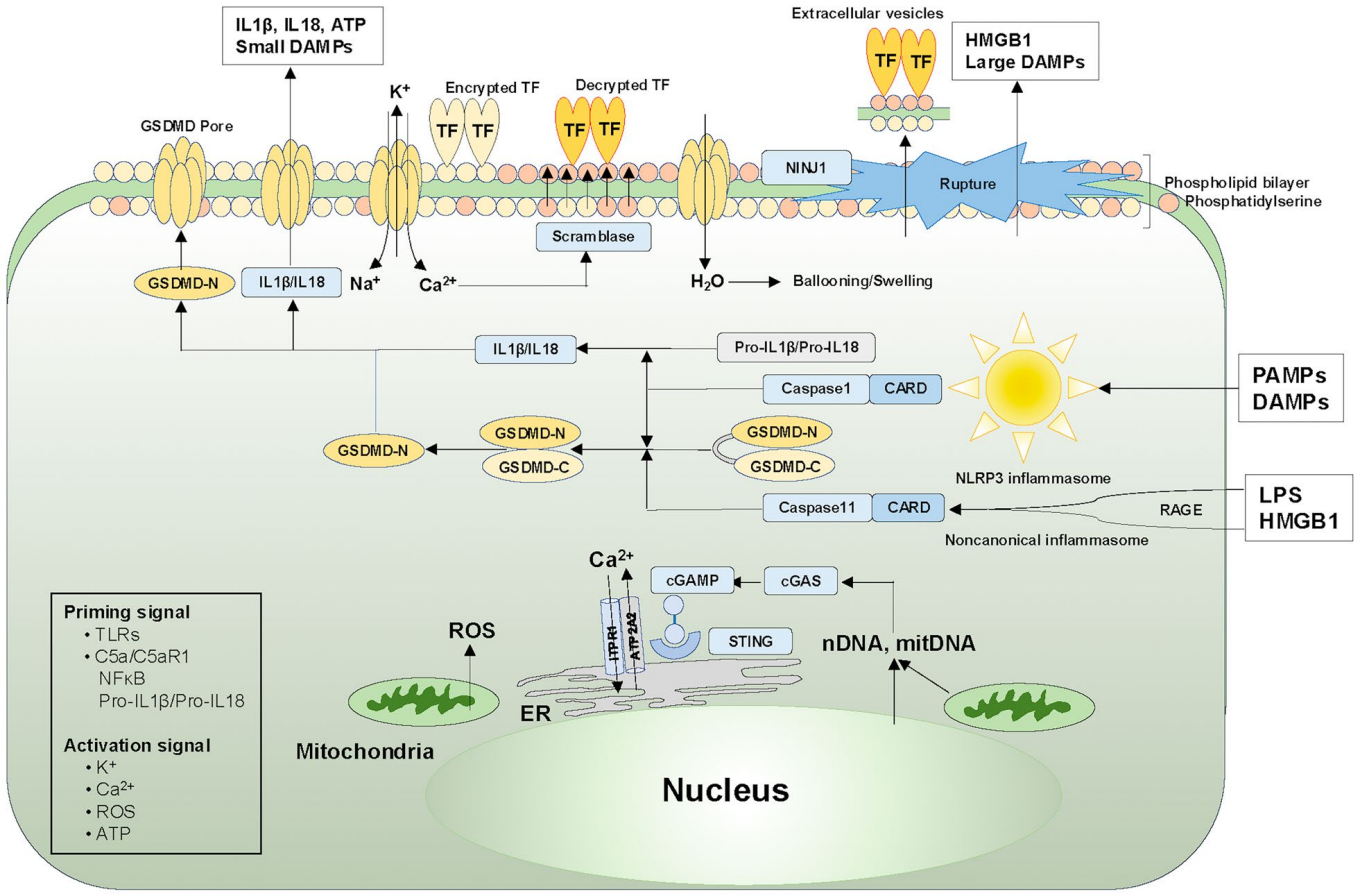
Pyroptosis triggers the release of tissue factor-positive extracellular vesicles. PAMPs and DAMPs activate the NLRP3 inflammasome through the NLR family NLRP3. LPS complexed with HMGB1 translocate to cells through RAGE, resulting in caspase11 (noncanonical inflammasome) activation. Two signals are required to activate the NLRP3 inflammasome. Transcriptional expressions of pro-IL1β and pro-IL18 via NFκB activation by PAMP and DAMP recognition via TLRs or C5a/C5aR act as priming signals. The second signals are K^+^ efflux, Ca^2+^ influx, ROS release from dysfunctional mitochondria, and ATP influx that activate inflammasomes. Canonical and noncanonical inflammasomes with activated caspases 1 and 11, respectively, cleave GSDMD to produce GSDMD-N, forming GSDMD-N pores, which release IL1β, IL18, and small DAMPs. The pore allows Ca^2+^, Na^+^, and H_2_O influx and K^+^ efflux. The Ca^2+^ activates phospholipid scramblase, exposing phosphatidylserine to the outer membrane, which changes tissue factors from encrypted to decrypted forms. Water-induced cell ballooning and swelling are followed by NINJ1-dependent plasma membrane rupture, releasing tissue factor-positive extracellular vesicles, HMGB1, and large DAMPs. Cytosolic DNA sensor cGAS/cGAMP/STING-induced Ca^2+^ release from ER is triggered by mitDNA and nDNA from dysfunctional mitochondria or nuclei. The dysfunctional mitochondria also release ROS. Both Ca^2+^ and ROS activate inflammasome caspases. ATP, adenosine triphosphate; ATP2A2, ATPase sarcoplasmic/endoplasmic reticulum Ca^2+^ transporting protein 2; *CARD* caspase activation recruitment domain, *cGAMP* cyclic GMP–AMP, *cGAS* cyclic GMP–AMP synthase, *DAMPs* damage-associated molecular patterns, *ER* endoplasmic reticulum, *GSDMD* gasdermin D, *HMGB1* high-mobility group box-1, *ITPR1* inositol 1,4,5-trisphosphate receptor type 1, *IL* interleukin, *LPS* lipopolysaccharide, *mitDNA* mitochondria DNA, *nDNA* nuclear DNA, *NFκB* nuclear factor-κB, *NLR* NOD-like receptor, *NLRP3* NLR pyrin domain-containing protein3, *NOD* nucleotide-binding oligomerization domain, *NINJ1* nerve injury-induced protein 1, *PAMPs* pathogen-associated molecular patterns, *RAGE* receptor for advanced glycan end-products, *ROS* reactive oxygen species, *STING* stimulator of interferon gene, *TF* tissue factor, *TLR* toll-like receptor

A cytosolic DNA sensor, cyclic GMP–AMP synthase (cGAS), along with its second messenger, cyclic GMP–AMP (cGAMP), binds to its adaptor molecule, stimulator of interferon gene (STING), which is expressed on the endoplasmic reticulum (ER), activating inflammasome-induced pyroptosis [100]. STING activation drives cytosolic calcium increases from the ER to activate caspases cleaving GSDMD, followed by pyroptosis and tissue factor release in monocytes and macrophages [104]. This study showed that STING pathway upregulation correlates with DIC severity, MODS, and mortality in murine sepsis models. Figure 3 shows the role of cell death in tissue factor release.

#### DAMPs

NET-derived DNA enhances thrombin generation through the intrinsic coagulation pathway [105]. An in vitro study showed that nDNA purified from human neutrophils activates the FXII-dependent coagulation pathway by amplifying tissue factor-mediated thrombin generation and thrombin-dependent FXI activation [106]. Cell-free RNA derived from damaged and necrotic cells also triggers FXI and FXII activation [107]. The histone–DNA complex, a marker of NET formation, determines FXIIa levels in circulation, which are associated with poor prognosis in patients with DIC [108]. Mitochondrial stress and necrosis release mitDNA into the cytosol and systemic circulation, resulting in systemic inflammatory response syndrome (SIRS) and organ injury [109]. Cytosolic mitDNA activates the NLRP3 inflammasome or the cGAS/cGAMP/STING pathway [110], potentially triggering pyroptosis and tissue factor release. Furthermore, the mitDNA-stimulated cGAS/cGAMP/STING pathway activation in neutrophils is associated with increased production of neutrophil elastase and nDNA derived from NETs [111]. These studies suggest that both nDNA and mitDNA are involved in tissue factor release, thrombin generation, and inflammation via diverse mechanisms to contribute to the development of DIC with SIRS.

The non-histone–chromatin nuclear peptide HMGB1 is released from the nucleus into circulation both passively during necrosis and actively during pyroptosis from monocytes and macrophages, evoking proinflammatory cytokine production [96, 112, 113]. Moreover, HMGB1 carries lipopolysaccharide (LPS) into cells via its receptor for advanced glycan end-products (RAGE), resulting in caspase-11 (noncanonical inflammasome) activation, followed by GSDMD cleavage and pyroptosis, associated with tissue factor release from macrophages [114, 115]. Furthermore, HMGB1 reinforces its procoagulant effects through NET formation and platelet activation via RAGE, leading to the release of monocyte-derived tissue factors and proinflammatory cytokines [116–118]. Platelet-derived HMGB1 induces platelet activation, aggregation, and thrombosis via the TLR4 signaling pathway [119]. HMGB1 accelerates inflammation and coagulation, resulting in glomerular fibrin deposition and alveolar hemorrhage, exacerbating renal and lung injuries and mortality in a thrombin-induced DIC model [118]. These results were clinically confirmed by the correlation between HMGB1 levels and DIC and sequential organ failure assessment (SOFA) scores in patients with various underlying DIC conditions [120].

### Hypoxia

Interactions between hypoxia, inflammation, and coagulation via hypoxia-inducible factor (HIF) have emerged as key contributors to DIC pathogenesis. Although multiple transcription factors respond to hypoxia, a main among these is HIF comprising oxygen-dependent HIFα and constitutively expressed HIFβ subunits, with the two major isoforms being HIF1 and HIF2 [121]. In hypoxia, HIFα translocates to the nucleus and recruits HIFβ, binding to hypoxic responsive element (HRE) and p300/cytoplasmic polyadenylation element binding protein (CREB)-binding protein (CBP), which leads to the transcriptional expression of hypoxic response genes related to inflammation, coagulation, and angiogenesis [121–123]. Pathological hypoxia in infected and ischemic tissues due to increased oxygen consumption, decreased oxygen delivery, or reduced vascularization leads to dysregulated immune inflammation, contributing to disease progression and organ dysfunction [121]. Typical hypoxic clinical conditions include sepsis with increased HIF activity [124], cardiac arrest, shock from various causes, such as trauma [125, 126], and tumor microenvironments [127]. These conditions significantly contribute to DIC development.

#### Inflammation and coagulation

In a rat model of hypoxia-induced venous thrombosis, hypoxia accelerated thrombosis via NLRP3 inflammasome activation and increased interleukin1β (IL1β) secretion [128]. Hypoxic thrombosis was associated with increased levels of prothrombin fragment 1 + 2, D-dimer, and PAI-1, along with increased t-PA levels, which correspond to DIC with a fibrinolytic phenotype [31, 128]. Caspase1 inhibition and NLRP3 or HIFα knockdown reduced inflammasome activation and thrombosis under hypoxic conditions, suggesting associations between HIFα and the NLRP3 inflammasome–caspase1–IL1β signaling pathway [128]. Hypoxia upregulates TLR4 expression in macrophages and expresses tumor necrosis factor α (TNFα) and IL6 via HIF1α [123, 129]. HIF1α also induces tissue factor [130] and PAI-1 [131] expression, and reduces protein S expression[132], while HIF2α downregulates TFPI expression [133]. Furthermore, TNFα and IL1β drive nuclear factor-κB (NFκB), promoting HIF1α mRNA transcription at hypoxic inflammation sites [121, 122].

**Fig. 4:**
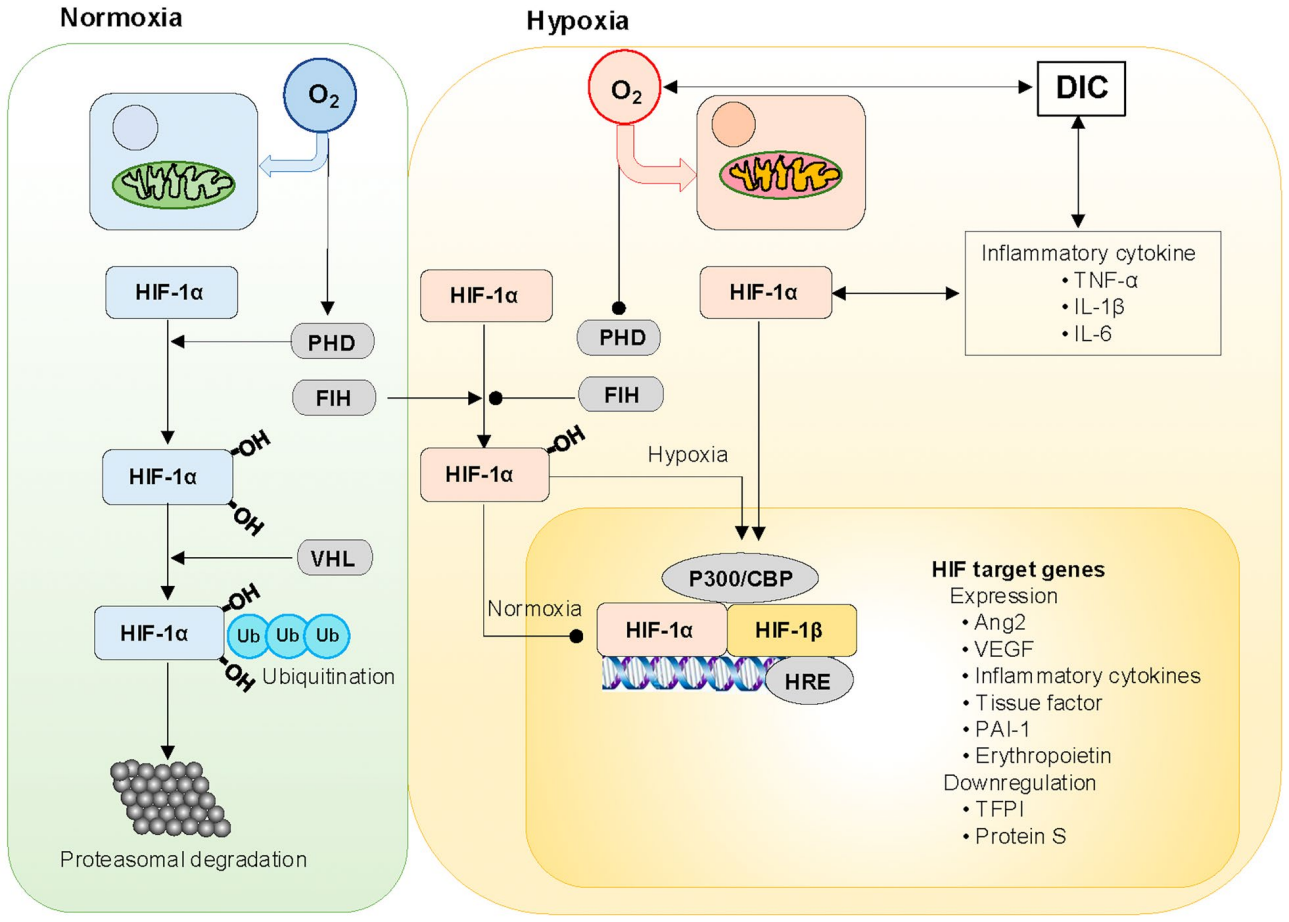
Bidirectional interplays among hypoxia, inflammation, and coagulation. Normoxia: HIF-1α hydroxylation by PHD and FIH is facilitated by spare non-mitochondrial oxygen, leading to ubiquitination by VHL, which results in proteasomal degradation and transcriptional suppression by FIH, preventing HIF-1α binding to transcriptional co-activator protein p300/CBP in the nucleus. Hypoxia: absence of spare oxygen inhibits HIF-1α hydroxylation by PHD and FIH, resulting in its translocation to the nucleus to recruit HIF-1β and binding to p300/CBP and HRE. This transcriptional complex leads to the expression or downregulation of HIF-1α target genes, including inflammatory cytokines. One of the main pathogeneses of DIC, inflammatory cytokines, conversely induce HIF-1α expression. Tissue hypoxia by DIC enhances HIF-1α translocation to the nucleus. Therefore, bidirectional interplays exist among hypoxia, inflammation, and coagulation, playing pivotal roles in DIC pathogenesis. *Ang* angiopoietin, *CBP* CREB-binding protein, *CREB* cytoplasmic polyadenylation element binding protein, *DIC* disseminated intravascular coagulation, *FIH* factors inhibiting HIF, *HIF* hypoxia-inducible factor, *HRE* hypoxic responsive element, *IL* interleukin, *PAI-1* plasminogen activator inhibitor 1, *PHD* prolyl hydroxylase, *TFPI* tissue factor pathway inhibitor, *TNF* tumor necrosis factor, *Ub* ubiquitin, *VEGF* vascular endothelial growth factor, *VHL* von Hippel–Lindau protein

These studies show that hypoxia-induced HIF may be involved in DIC pathogenesis via increased inflammation and coagulation, anticoagulation impairment, and fibrinolysis suppression. Conversely, proinflammatory cytokine-induced SIRS and secondary hypoxic changes in tissues due to microvascular thrombosis are also DIC features [134]. Therefore, it is plausible that hypoxia, inflammation, and DIC are linked (Fig. 4).

#### Angiopoietin and Tie2

Hypoxic tissues require blood supply and angiogenesis is stimulated. HIF1α targets angiogenic factors, such as angiopoietin2 (Ang2) and vascular endothelial growth factor (VEGF) [135, 136]. Ang2 and constitutively expressed Ang1 have antagonistic and agonistic effects on their endothelial receptor, Tie2, respectively. Moreover, their balance maintains antithrombotic, anti-inflammatory, and non-angiogenic vascular quiescence and permeability [135]. However, Ang2 expression induction by HIF1α and proinflammatory cytokines and Ang2 release from its storage pool of Weibel–Palade bodies due to hypoxia and thrombin can antagonize Ang1-mediated anti-inflammatory and anti-thrombotic milieu of endothelial cells [135, 137]. Ang1 antagonizes VEGF-induced expressions of E-selectin, intercellular adhesion molecules, vascular cell adhesion molecules, and tissue factors, which are also overwhelmed by increased Ang2 [135, 138, 139]. Furthermore, Ang1/Tie2 maintains endothelial permeability, which is abrogated by Ang2 and VEGF action on VEGF receptor2 [135, 140].

In LPS-induced endotoxemia, Ang/Tie2 axis disruption precedes signs of DIC and recapitulates sepsis-related thrombus formation, whereas Tie2 activation suppresses LPS-mediated prothrombotic actions on the endothelium [141]. Ang2 heterozygous mice showed reduced vascular inflammation and organ dysfunction, and Ang2 inhibition attenuated endothelial injury [142]. These two studies implicate that Ang2/Tie2 signaling in regulating microvascular thrombosis in septic DIC [141]. Clinical studies support this notion, showing high Ang2 levels and Ang2/Ang1 ratios, as well as associations between Ang2 levels and the development of MODS and mortality in patients with DIC [141–145]. Although the role of VEGF in DIC has not been extensively studied, in vitro studies showed that Ang2 inhibition prevented septic shock serum-induced endothelial paracellular gap formation, implying that the Ang/Tie2 and VEGF systems contribute to capillary leakage in DIC [142].

### KKS and complement pathway

#### Bidirectional interplay

**Fig. 5:**
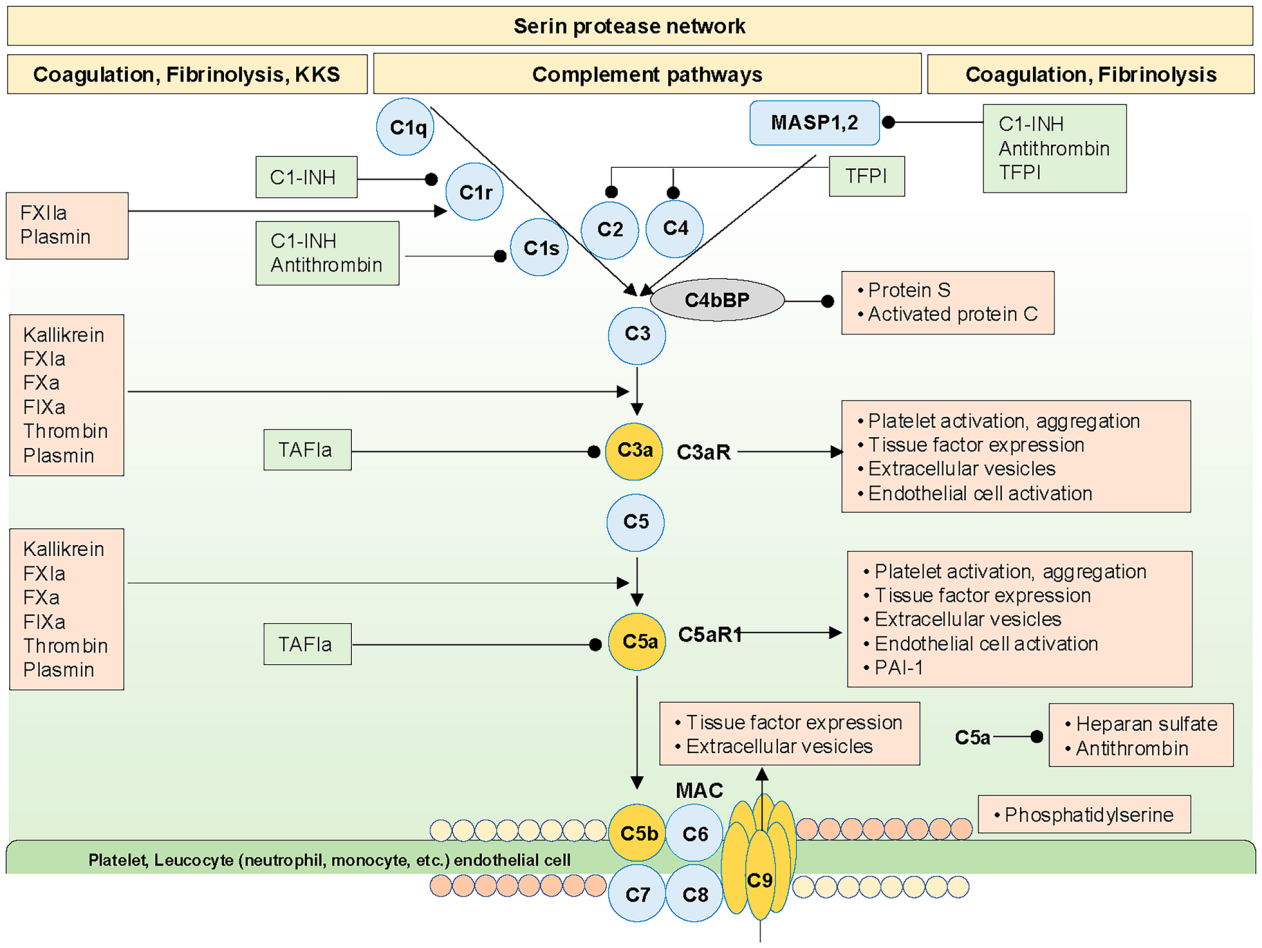
Serine protease network and its inhibitory mechanisms. Activated coagulation factors, thrombin, plasmin, and kallikrein noncanonically activate complements, which in turn activate platelets, coagulation, and endothelial cells associated with fibrinolysis suppression by PAI-1 through C3/C3aR and C5/C5aR1. Anticoagulation pathways are also dampened by C4bBP binding to protein S and C5a-induced glycocalyx degradation and shedding, reducing activated protein C and antithrombin functions, respectively. MAC-injured plasma membrane releases tissue factor-bearing extracellular vesicles and increased intracellular Ca^2+^ exposes phosphatidylserine on the outer membrane by scramblase activation. These processes are controlled by C1-INH, antithrombin, TFPI, and TAFIa, as depicted in the figure. Furthermore, antithrombin indirectly controls complement pathways by inhibiting kallikrein, factors XIIa, XIa, Xa, and IXa, and thrombin. Dysregulation of these control mechanisms in severe insults can easily progress to DIC. *C1-INH* C1 esterase inhibitor, *C4BP* C4b binding protein, *KKS* Kallikrein–Kinin system, *MAC* membrane attack complex, *MASP* mannose-binding lectin-associated serine protease, *TAFIa* activated thrombin-activatable fibrinolysis inhibitor, *TFPI* tissue factor pathway inhibitor

Coagulation, KKS, and the complement pathways constituting the serine protease network are involved in DIC pathogenesis. Various DAMPs (DNA, RNA, and polyphosphate) activate FXII to FXIIa, initiating KKS, followed by bradykinin production, which is associated with increased fibrinolysis and inflammation [83, 105, 107]. The detailed relationships between KKS, fibrinolysis, inflammation, and DIC have been previously reviewed [31, 67]. The bidirectional interplay between coagulation, KKS, and the complement pathway has also been extensively discussed and summarized in Figs. 1 and 5 [14, 67, 68, 146].

Briefly, FXIIa, FXIa, FXa, FIXa, thrombin, kallikrein, and plasmin act on C1r, C3, and C5 to form C3a, C5a, and C5b-9 complexes (membrane attack complex, MAC). These complements can induce platelet and coagulation activation, anticoagulation system impairment, fibrinolysis suppression, and endothelial injury. Conversely, complement factor H (CFH) binds to platelets, regulating their activation and aggregation. In addition, CFH directly inhibits FXIIa and FXIa, and enhances the thrombin–thrombomodulin complex-induced protein C activation, controlling thrombin generation by inhibiting FVa and FVIIIa [147]. Both thrombin and plasmin directly activate C3 and C5 in vivo and in vitro [148, 149]. Furthermore, they may act on C3 and C5 through a synergistic action with LPS or bacteria in nonhuman primates [150]. Although the crosstalk between the coagulation and complement pathways is an innate defense mechanism against insults, dysregulation (such as in sepsis) can lead to DIC and organ dysfunction [151, 152].

#### Complement inflammasome axis

Three complement pathways recognize DAMPs and PAMPs as triggers, converging to form C3a, C5a, and MAC, which evoke inflammation via receptor activation or pore formation [153]. C5a/C5a receptor1 (C5aR1) signaling activates NFκB and activator protein 1, inducing proinflammatory cytokine expression. In addition, C5a induces IL1β gene transcription and releases TNFα and IL1β into circulation during DAMP-triggered complement pathway activation [154]. Following these priming, C3a/C3aR induces adenosine triphosphate (ATP) efflux, activating purinoreceptors [155], while C5a/C5aR1 stimulates mitochondrial reactive oxygen species (ROS) release [155, 156], leading to NLRP3 inflammasome activation and IL1β secretion. C5aR2 immunomodulates C5aR1 response, regulating C5aR1-mediated proinflammatory signaling [153] while also contributing to NLRP3 inflammasome activation and HMGB1 release [157]. HMGB1, which is a large DAMP, is usually released through membrane rupture via pyroptosis [96], suggesting that pyroptosis plays a role in C5a-induced tissue factor release and DIC development [101, 102]. MAC also activates the NLRP3 inflammasome and IL1β secretion via ATP influx by K^+^ efflux and Ca^2+^ influx through MAC-formed pores and, in part, Ca^2+^ release from the ER [158, 159]. Internalized MAC leads to inflammasome assembly and activates the NLRP3 inflammasome independently of ion flux [160, 161]. C3a-, C5a-, and MAC-induced NLRP3 inflammasome activation may be associated with pyroptosis, which may play a role in DIC pathogenesis. The complement and inflammasome axis is comprehensively reviewed elsewhere for further study [162, 163].

#### NETosis and complement pathways

Mutual interactions between NETosis and complement pathways form a triangular relationship with coagulation [164]. NETosis is tightly regulated and requires C3, C3aR, or C3b-dependent opsonization [165, 166]. Anaphylatoxin C5a is also involved in NETosis. Priming neutrophils with C5a enhances antibody-induced NETosis; further, C5a alone stimulates NETosis when interferons α and γ are used for priming [167, 168]. In addition to C3, CFB, and properdin expression in neutrophils, released NETs also deposit three factors required for C3 convertase formation in an alternative pathway [169, 170]. NETs also deposit CFH, which inhibits and degrades C3b. The co-deposition of CFB and CFH suggests a balance between complement activation and inhibition on NETs [171]. Therefore, NETs provide a platform for complement activation and probably for complement interaction with histones and coagulation factors to induce immunothrombosis.

The discussed DIC pathogenic pathways are primarily innate immune reactions to immunothrombosis at the site of insult. However, if dysregulated, these reactions progress to DIC, resulting in organ dysfunction, irrespective of infectious or non-infectious insults [10, 172].

## Organ dysfunction

Recent advances have deepened the understanding of organ dysfunction pathogenesis in DIC, where DIC and DAMPs act as pathogenic triggers, synergistically leading to MODS and poor patient prognoses [1, 13, 54]. In DIC, systemic microvascular thrombosis reduces oxygen delivery to peripheral tissues, resulting in ischemic organ dysfunction. Inflammation-induced endothelial injury, glycocalyx degradation, and mitochondrial dysfunction cause tissue dysoxia, where oxygen extraction fails to meet increased cellular demand despite normal oxygen delivery, giving rise to inflammatory organ dysfunction. Excessive hemorrhage in DIC may accelerate ischemic and inflammatory organ dysfunction through tissue hypoxia due to anemia and hypoperfusion.

### DAMPs

#### Histones and NETs

Histones and NETs play central roles in MODS pathogenesis in DIC. Together, they invoke organ dysfunctions in the brain, heart, lungs, liver, kidney, and pancreas [173]. Histones binding to anionic phospholipids cause direct cellular injury through cell integrity disruption and large calcium influx in vitro [72, 174, 175], which is associated with reduced cardiomyocyte viability and contractility [174]. In in vivo models, histone infusion resulted in left ventricular dysfunction [174]; increased plasma levels of cell-specific damage markers in the liver, kidney, heart, and endothelial cells; and worsened lung injury scores [72, 175]. Histones also impair right ventricular function, probably owing to increases in afterload due to pulmonary microvascular obstruction by neutrophil congestion, NETosis, and fibrin thrombosis [95, 174]. Notably, in vivo, histone infusion reproduces DIC with thrombosis in vital organs [79, 95]. Clinically, high levels of circulating histones are associated with lung injury and elevated SOFA scores in trauma and sepsis, with patients with septic shock and/or DIC having higher histone levels and SOFA scores than those without these conditions [72, 95, 175].

In vitro, NETs may directly induce hepatocyte death [176]; however, a study showing NET-mediated histone deposition in endothelial and epithelial cell death highlighted a significant role of histones in NET-mediated cell death [177]. In addition, a clinical study that directly measured NET formation capacity identified NET formation as an independent predictor of DIC and mortality. The degree of NET formation significantly correlated with DIC and SOFA scores in critically ill patients [178].

#### MitDNA and HMGB1

MitDNA and HMGB1 are also involved in organ dysfunction. Thousand-fold higher mitDNA levels were observed immediately after trauma [109]. In this study, mitDNA accelerated neutrophil Ca^2^^+^ influx and activation in vitro, with activated neutrophil signaling inducing lung injury with TNFα and IL6 accumulation in a mitDNA infusion model. In vitro studies showed that cardiac myocytes co-incubated with HMGB1 had reduced cell viability associated with changes in calcium handling and a substantial decrease in mitochondrial respiratory capacity [179]. Lung and kidney injury exacerbation by HMGB1 was observed in a thrombin-induced rat DIC model [118]. Moreover, in trauma and ischemia–reperfusion models, HMGB1 neutralization or inhibition prevented hepatocyte injury [180, 181]. The damaging effects of HMGB1 on endothelial cells result in endothelial inflammation, endothelial integrity loss, and endothelial injury, suggesting the involvement of endothelial cells in HMGB1-induced organ dysfunction [182, 183]. A clinical study of patients with various underlying DIC disorders showed high HMGB1 levels in patients with DIC or organ dysfunction, which significantly correlated with DIC and SOFA scores [120].

### Complement pathway

About two decades ago, the harmful effects of the complement pathway on organ function were acknowledged in sepsis [184, 185]. Since then, its mechanisms have become increasingly understood beyond sepsis, extending to other insults. The C5a/C5aR axis and MAC play crucial roles in complement-mediated MODS, which have been extensively reviewed elsewhere [186–188]. In summary, activated and dysregulated complement pathways induce tissue factor expression, resulting in DIC and organ dysfunction in the heart, lungs, and kidneys, including endothelial injury and glycocalyx disruption [184, 187]. Proposed pathological mechanisms include C5a/C5aR-induced histone release via NETs from neutrophils and macrophages, MAC-induced Ca^2+^ influx in epithelial and endothelial cells causing mitochondrial dysfunction, and NLRP3 inflammasome activation by both C5a/C5aR and MAC, resulting in IL1β secretion [188].

## Management

No randomized controlled trial has demonstrated the utility of a single drug for DIC treatment; however, considerable progress has been made since the publication of DIC treatment guidelines by the ISTH [189]. Using a systematic review and meta-analysis, the Japanese Society of Thrombosis and Hemostasis (JSTH) also published guidelines for DIC across various underlying disorders [190] (1 in 8 guidelines are cited).

Effective worldwide DIC management relies on aggressive treatment of the underlying disorder. Although treatment thresholds and types of concentrate may differ, substitution therapies are universally applied for consumptive hemorrhage in DIC. Tranexamic acid has been used for critical bleeding in trauma and postpartum complications [191, 192]. Although the study participants were not patients with DIC, tranexamic acid may be permitted for early stage DIC with a fibrinolytic phenotype following trauma and parturition [189, 190]. However, DIC management usually involves anticoagulant therapy, which will be discussed below, with reference to sepsis-induced DIC.

### Rationale

#### Why

Innate immune inflammation and coagulation at the site of insult form immunothrombosis, restricting and fixing insults locally and maintaining homeostasis and recovery. However, if the insult is sufficiently severe, dysregulated innate immunity results in DIC with systemic thrombosis and inflammation, leading to MODS associated with death [10, 172]. Therefore, to improve patient outcomes, management of not only the underlying disease but also DIC while providing organ support is necessary [1].

#### To whom

Except for the PROWESS trial, all randomized controlled trials of anticoagulants for sepsis or sepsis with coagulopathy aiming at reducing mortality failed [193–196]. Ultimately, PROWESS also failed to obtain significant results, leading to the withdrawal of its trial drug from the market [197, 198]. However, post hoc analyses of PROWESS and KyberSept trials have shown promising results for recombinant human-activated protein C (rhAPC) and antithrombin use in patients with sepsis and DIC [199–201]. In addition, a post hoc analysis of the SCARLET trial showed that the recombinant human thrombomodulin (rhTM) group with higher baseline thrombin generation marker levels, that mimics DIC, demonstrated lower mortality than the placebo group [202]. Although debates on anticoagulant use and the need for more robust data should be fully considered [203, 204], a meta-analysis of randomized controlled trials suggests that the target population of anticoagulants are patients with confirmed DIC, not those with sepsis or sepsis and coagulopathy [205].

Based on a subgroup analysis of the PROWESS trial, the Food and Drug Administration approved rhAPC for patients with sepsis with an Acute Physiology and Chronic Health Evaluation (APACHE) II score > 25 [206]. In contrast, the ADDRESS trial found no benefit of rhAPC in patients with sepsis and APACHE II score < 25 [207], suggesting that disease severity is inferred from rhAPC use in sepsis [197]. Subgroup analyses of the KyberSept trial for antithrombin showed an increased survival probability in patients with sepsis, with a predicted 30–60% mortality at study entry according to the Simplified Acute Physiology Score II [208]. Similar results were obtained with rhTM, showing improved survival probability in patients with sepsis-induced DIC with a 24–29 APACHE II score [209]. A large nationwide registry study showed the survival benefit of anticoagulant therapy in patients with sepsis-induced DIC and/or SOFA scores between 13 and 17 [210]. In summary, the optimal target of anticoagulant therapy for sepsis may be patients with DIC and high disease severity, requiring future randomized controlled studies [211].

#### When

A peculiar finding from the PROWESS trial showing significantly lower mortality in placebo-treated patients with the prothrombotic FV Leiden heterozygous polymorphism than in placebo-treated non-FV Leiden patients (31.0% vs. 15.6%) was confirmed in a murine endotoxemia model [212]. The results showed that adequate thrombin generation is necessary at the early stages of endotoxemia; however, persistently large amounts of thrombin generation at later stages are deleterious to mouse survival, suggesting that sufficient thrombin may be required for immunothrombosis at an early stage of the insult. However, continuous thrombin generation may produce thrombotic organ dysfunction, leading to death. Excessive fibrinolysis by the Pla protease of *Yersinia pestis* disrupts fibrin deposition, concentrating bacteria without inflammatory cells at the infection site, leading to low rabbit survival [213]. This study also suggests the importance of robust immunothrombosis formation at the site of the insult. In clinical settings, immunothrombosis progressing to systemic thrombosis and inflammation can be recognized by established DIC diagnosis. Therefore, DIC treatment should be initiated only after a definite DIC diagnosis to avoid disrupting immunothrombosis.

#### How

**Fig. 6:**
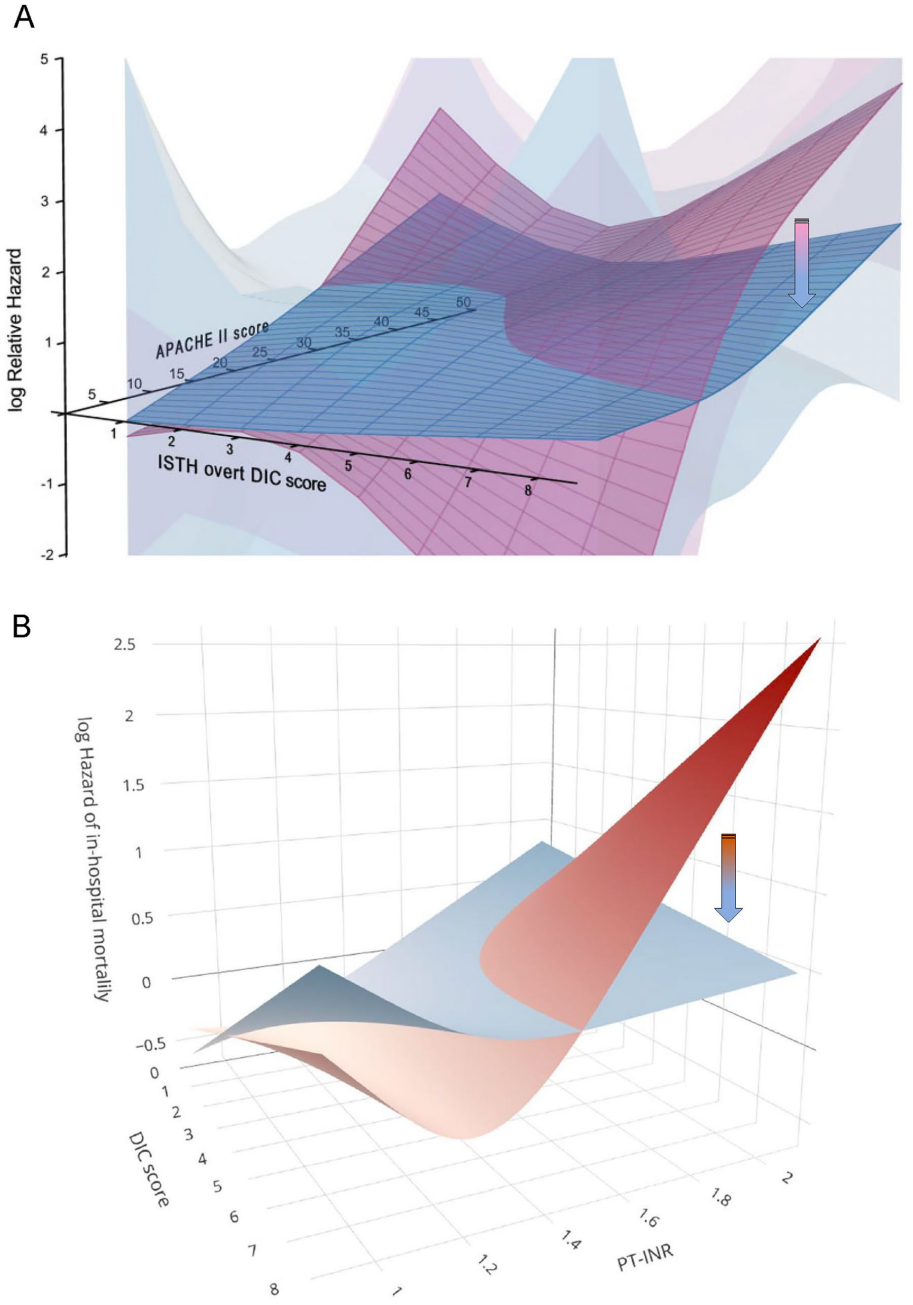
Hazard of in-hospital mortality by DIC score and disease severity. The optimal target population for anticoagulant therapy in sepsis is not all patients with sepsis or sepsis-induced coagulopathy, but specifically those with an established DIC diagnosis and high disease severity. **A** The arrow indicates that anticoagulant therapy (blue plate) decreased the relative hazard ratio for in-hospital mortality compared with the control (non-anticoagulant; pink plate) in patients with sepsis diagnosed as having DIC and high APACHE II scores (shaded areas indicate 95% confidence interval). Reprint permission (Georg Thieme Verlag KG, No.5965741147872. Feb 11, 2025). **B** Anticoagulant therapy (blue plate) decreased hazard ratio of in-hospital mortality compared with the control (non-anticoagulant; red plate) in patients with higher JAAM DIC score and PTINR ≥ 1.5 in patients with sepsis. Reprint permission under a Creative Commons Attribution 4.0 International License. *APACHE* Acute Physiology and Chronic Health Evaluation, *DIC* disseminated intravascular coagulation, *ISTH* International Society on Thrombosis and Hemostasis, *JAAM* Japanese Association for Acute Medicine, *PTINR* prothrombin time international normalized ratio

Multivariate Cox proportional regression analysis in sepsis showed significant interactions between anticoagulation therapy, ISTH DIC score, and APACHE II score, confirming a marked reduction in the hazard ratio of in-hospital mortality by anticoagulant therapy in patients simultaneously having ISTH DIC and high APACHE II score [214]. These results suggest the usefulness of monitoring ISTH DIC and APACHE II scores in selecting optimal patients for anticoagulant therapy. A study using the prothrombin time international normalized ratio (PTINR) as a substitute for SOFA scores showed that the hazard ratio for in-hospital death decreased in patients with definite JAAM DIC (score ≥ 5) and PTINR ≥ 1.5, indicating an optimal threshold of these parameters for selecting anticoagulant therapy in patients with sepsis [215]. Monitoring DIC scores and risk stratification based on severity scores are crucial in clinical settings. The results are shown in Fig. 6.

#### Drugs

**Fig. 7:**
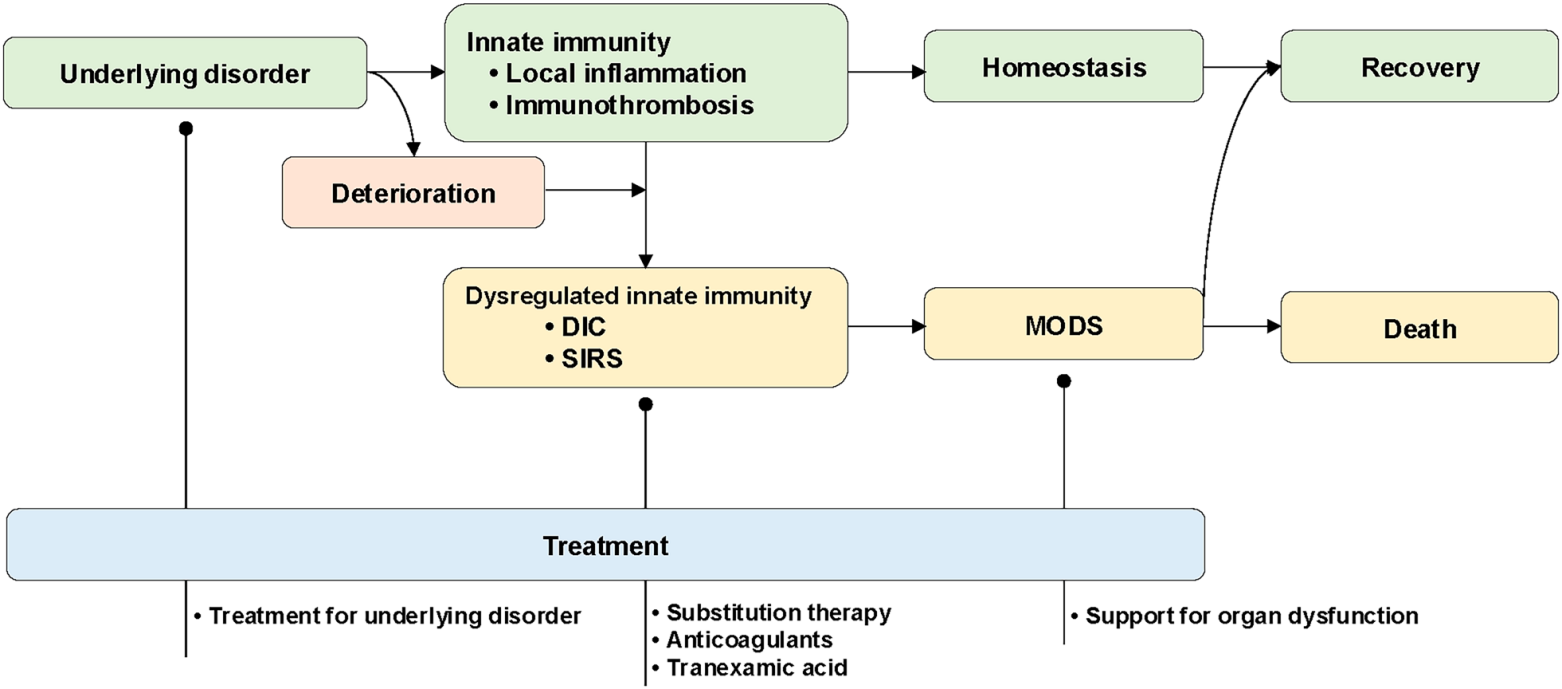
Diverse disorders evoke innate immunity comprising local inflammation associated with immunothrombosis at the site of insults, which aims to maintain homeostasis, leading to recovery. When the insults are severe and persistent, dysregulated innate immunity cannot restrict the insults locally, developing into disseminated immunothrombosis with systemic inflammation, which is defined as DIC, which can lead to MODS and death of the patient. In addition to treating underlying DIC disorders, DIC treatments with systemic inflammation and organ dysfunction are mandatory to improve patient outcomes. *DIC* disseminated intravascular coagulation, *MODS* multiple organ dysfunction syndrome, *SIRS* systemic inflammatory response syndrome

Although antithrombin and rhTM are available only in certain regions for the treatment of DIC, and no randomized controlled trials have confirmed their efficacy, both the JSTH and the Japanese Society of Intensive Care Medicine (JSICM) support their use for sepsis-induced DIC, providing strong or weak recommendations based on a moderate level of evidence (GRADE 2B) [190, 216]. Unfractionated heparin and low molecular weight heparin are commonly used for DIC treatment. However, a high level of evidence is currently lacking in the efficacy of the two drugs for the DIC treatment in sepsis. The JSTH has not issued a clear recommendation, and the JSICM has designated these drugs as a subject for future research question. Management strategies are summarized in Fig. 7.

### Future

Many studies have illustrated the promising potential of the therapeutic interventions targeting histones and NETs. In addition to the promising effects of anti-histone antibodies that neutralized histones [72, 78, 174], rhTM and non-anticoagulant heparin bind to histones and prevent prothrombotic and cytotoxic effects of histones, respectively [95, 217]. Three neutrophil serine proteases, neutrophil elastase, cathepsin G and proteinase 3, cleave histones [218]. Although the causal effects of histone cleavage by these proteases on anticoagulation and cytoprotection have not been confirmed, further studies are warranted. APC is also known to bind and proteolyze histones, thereby reducing their cytotoxic effects [9, 219]. However, the anticoagulant effect limits its potential for cytoprotection. To address this, optimized APC variants have been developed by computer-aided molecular design method, which may be applied to histone-mediated DIC [219]. There is also potential for therapeutic approaches targeting NETs, including DNase, peptidyl arginine deiminase type IV, and ADAMTS13 [8, 220, 221].

## Conclusions and future perspective

This review explores DIC pathophysiology from the perspective of innate immune reactions against insults based on the latest findings. Over the past half-century, DIC research has substantially progressed, especially in how thrombin, a key player in DIC, is generated in response to insults. Histones seem to play crucial roles in increasing thrombin generation in DIC and subsequent organ dysfunction, suggesting that targeting histones may become a specific and effective therapeutic strategy in DIC management. Future studies should focus on developing, standardizing, and simplifying histone measurement techniques and other promising DIC diagnostic markers. Moreover, it is necessary to avoid past failures in megatrials targeting incorrect populations and to select optimal patients for prospective randomized controlled trials of DIC management.

## Data Availability

No datasets were generated or analysed during the current study.

## Abbreviations

ADAMTS-13: A disintegrin-like metalloproteinase with thrombospondin type 1 motifs 13
Ang: Angiopoietin
APACHE: Acute Physiology and Chronic Health Evaluation
APC: Activated protein C
AP1: Activator protein 1
ATP: Adenosine triphosphate
ATP2A2: ATPase sarcoplasmic/endoplasmic reticulum Ca^2+^ transporting protein 2
C1-INH: C1 esterase inhibitor
C3aR: C3a receptor
C4BP: C4b binding protein
C5aR: C5a receptor
CARD: Caspase activation recruitment domain
CBP: CREB-binding protein
CREB: Cytoplasmic polyadenylation element binding protein
CDR: Central Denmark Region hospital laboratory database
CFH: Complement factor H
cGAMP: Cyclic GMP–AMP
cGAS: Cyclic GMP–AMP synthase
DABAK: Des-Arg^9^-bradykinin
DAMPs: Damage-associated molecular patterns
DIC: Disseminated intravascular coagulation
DPC: Japanese Diagnosis Procedure Combination inpatients database
EPCR: Endothelial protein C receptor
ER: Endoplasmic reticulum
F: Factor
FDP: Fibrin/fibrinogen degradation products
FIH: Factors inhibiting HIF
GSDMD: Gasdermin D
HELLP: Hemolysis, elevated liver enzyme, and low platelet count
HIF: Hypoxia-inducible factor
HMGB1: High-mobility group box-1
HMWK: High molecular weight kininogen
HRE: Hypoxic responsive element
ICAM-1: Intercellular adhesion molecule-1
ICD: International Classification of Disease Tenth Revision
ICU: Intensive care unit
ITPR1: Inositol 1,4,5-trisphosphate receptor type 1
IL: Interleukin
ISTH: International Society on Thrombosis and Haemostasis
JAAM: Japanese Association for Acute Medicine
JMHW: Japanese Ministry of Health and Welfare
JSICM: Japanese Society of Intensive Care Medicine
JSTH: Japanese Society of Thrombosis and Hemostasis
KBR: Bradykinin receptor
KKS: Kallikrein–Kinin system
LPS: Lipopolysaccharide
MAC: Membrane attack complex
MASP: Mannose-binding lectin-associated serine protease 1
mitDNA: Mitochondria DNA
MODS: Multiple organ dysfunction syndrome
NA: Not applicable.
nDNA: Nuclear DNA
NETs: Neutrophil extracellular traps
NFκB: Nuclear factor-κB
NLR: NOD-like receptor
NLRP3: NLR pyrin domain-containing protein3
NOD: Nucleotide-binding oligomerization domain
NINJ1: Nerve injury-induced protein 1
PAI-1: Plasminogen activator inhibitor 1
PAMPs: Pathogen-associated molecular patterns
PC: Protein C
PHD: Prolyl hydroxylase
PS: Protein S
PTINR: Prothrombin time international normalized ratio
RAGE: Receptor for advanced glycan end-products
rhAPC: Recombinant human activated protein C
rhTM: Recombinant human thrombomodulin
ROS: Reactive oxygen species
SIRS: Systemic inflammatory response syndrome
SOFA: Sequential organ failure assessment
STING: Stimulator of interferon gene
TAFI: Thrombin-activatable fibrinolysis inhibitor
TAFIa: Activated thrombin-activatable fibrinolysis inhibitor
TF: Tissue factor
TFPI: Tissue factor pathway inhibitor
TLR: Toll-like receptor
TNF: Tumor necrosis factor
t-PA: Tissue-type plasminogen activator
Ub: Ubiquitin
u-PA: Urokinase (urinary)-type plasminogen activator
VCAM-1: Vascular cell adhesion molecule-1
VEGF: Vascular endothelial growth factor
VHL: Von Hippel–Lindau protein
